# Saponins from Chinese Medicines as Anticancer Agents

**DOI:** 10.3390/molecules21101326

**Published:** 2016-10-05

**Authors:** Xiao-Huang Xu, Ting Li, Chi Man Vivienne Fong, Xiuping Chen, Xiao-Jia Chen, Yi-Tao Wang, Ming-Qing Huang, Jin-Jian Lu

**Affiliations:** 1 State Key Laboratory of Quality Research in Chinese Medicine, Institute of Chinese Medical Sciences, University of Macau, Macao, China; xuxiaohuang1125@gmail.com (X.-H.X.); liting19890717@msn.cn (T.L.); vivfong530@gmail.com (C.M.V.F.); xpchen@umac.mo (X.C.); xiaojiachen@umac.mo (X.-J.C.); ytwang@umac.mo (Y.-T.W.); 2 College of Pharmacy, Fujian University of Traditional Chinese Medicine, Fuzhou 350122, China

**Keywords:** triterpenoid saponins, steroid saponins, mechanism, target, cancer

## Abstract

Saponins are glycosides with triterpenoid or spirostane aglycones that demonstrate various pharmacological effects against mammalian diseases. To promote the research and development of anticancer agents from saponins, this review focuses on the anticancer properties of several typical naturally derived triterpenoid saponins (ginsenosides and saikosaponins) and steroid saponins (dioscin, polyphyllin, and timosaponin) isolated from Chinese medicines. These saponins exhibit in vitro and in vivo anticancer effects, such as anti-proliferation, anti-metastasis, anti-angiogenesis, anti-multidrug resistance, and autophagy regulation actions. In addition, related signaling pathways and target proteins involved in the anticancer effects of saponins are also summarized in this work.

## 1. Introduction

Natural compounds isolated from Chinese medicines represent a large reservoir of potential leads for drug discovery. We have previously summarized the anticancer activities and mechanisms of action of terpenoids [1], quinones [2], and alkaloids [3] which have shown promising medicinal properties. Some naturally derived compounds, such as taxol and vincristine, have long been widely used as anticancer agents. Saponins, another type of plant-derived secondary metabolites, are glycosides containing aglycones of triterpene sapogenins or steroidal sapogenins. Based on their aglycone, saponins are divided into two main types, namely, triterpenoid saponins and steroidal saponins. The former type mainly exists in plants from the *Araliaceae*, *Leguminosae*, *Polygalaceae* and *Campanulaceae* families, whereas the latter mainly exists in the *Dioscoreaceae*, *Liliaceae*, and *Scrophulariaceae*.

Saponins exert various pharmacological effects, including cardiovascular protective activity [4], anti-inflammatory [5], antiviral [6], and immunoregulatory effects [7]. Moreover, recent studies have reported that saponins demonstrate significant anticancer activity, such as anti-proliferation [8], anti-metastasis [9], anti-angiogenesis [10] and reversal of multi-drug resistance (MDR) effects [11] through mechanisms that include induction of apoptosis and promotion of cell-differentiation. They had also been reported to reduce the side-effects of radiotherapy and chemotherapy [12], suggesting that saponins are a promising prospect for anticancer research and development. Our group has worked on the anticancer effects and mechanisms of saponins such as ginsenosides and platycodin D, and we found that the reviews on the anticancer properties of saponins were still lacking. For this reason, this work summarizes the anticancer activities, as well as the involved mechanisms, of the saponins ginsenoside, saikosaponin, dioscin, polyphyllin, and timosaponin. This work will hopefully serve as a reference for further development of saponins as anticancer agents.

## 2. Triterpenoid Saponins

### 2.1. Ginsenosides

**Figure 1:**
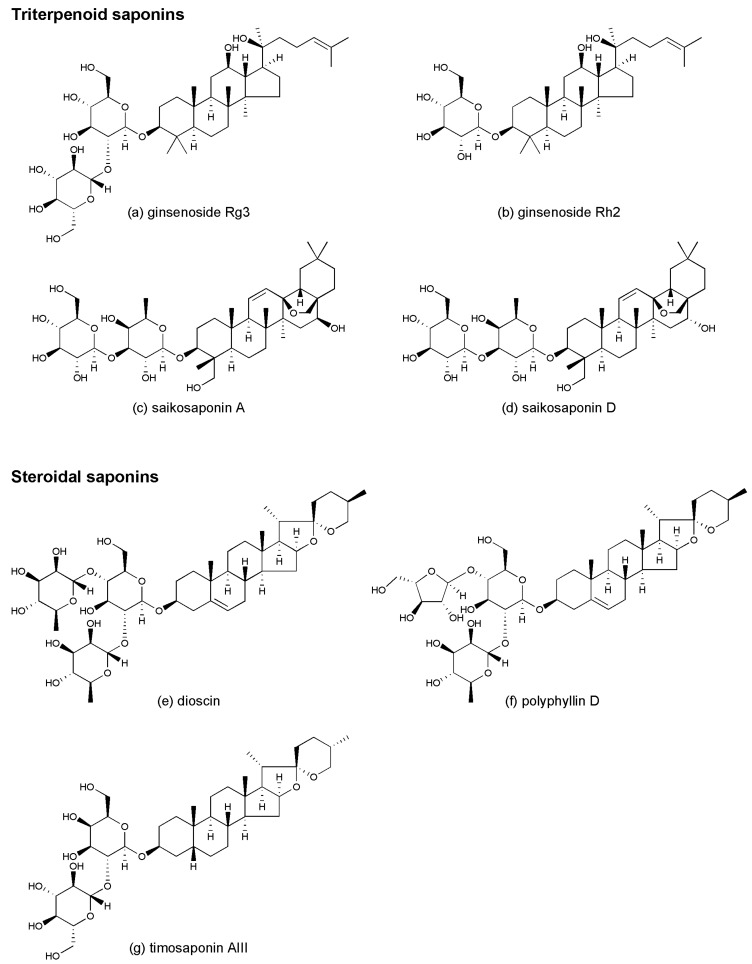
Chemical structures of saponins: (**a**) ginsenoside Rg3; (**b**) ginsenoside Rh2; (**c**) saikosaponin A; (**d**) saikosaponin D; (**e**) dioscin; (**f**) polyphyllin D; (**g**) timosaponin AIII.

Ginsenosides are the main active components of *Ginseng* *radix* (Renshen in Chinese), which is widespread in northeast China, Korea, and Japan. It has been used in Asian countries for centuries to treat various diseases because of its wide spectrum of pharmacological effects [13]. Additionally, ginsenosides are also found in American ginseng (Xiyangshen in Chinese). More than 100 ginsenosides have been isolated from *Ginseng radix* and are classified into protopanaxadiol (PPD), protopanaxatriol (PPT), ocotillol, and oleanolic acid types. The main structural difference between the PPD and PPT types is the presence of sugar residues attached to an α-OH at C-6 and the absence of β-OH at C-3 in the PPT moiety [14]. These compounds have recently received increasing attention due to their anticancer properties. Compared with the PPT-type ginsenosides, the PPD-type ginsenosides exhibit stronger anticancer potentials. Ginsenoside Rg3 (Figure 1a) and Rh2 (Figure 1b) are the most well-studied active PPD-type anticancer congeners. Other PPD-type ginsenosides, such as ginsenoside Rb1 [15], Rb2 [16], Rc [17], Rd [18], and Rk1 [19], as well as PPT-type ginsenosides such as Re [20] and Rf [21] have also demonstrated anticancer activities with similar mechanisms as those in ginsenoside Rg3 or Rh2. 

#### 2.1.1. Ginsenoside Rg3

Ginsenoside Rg3 demonstrates therapeutic effects in vitro and/or in vivo against various tumors, including leukemia [22], lung cancer [23], esophageal carcinoma [24], gastric cancer [25], colon cancer [26,27,28], hepatoma [29,30], renal cancer [31], bladder cancer [32], breast cancer [33], ovarian cancer [34], prostate cancer [35,36] and melanoma [37,38,39]. Moreover, combination therapy of ginsenoside Rg3 with other chemotherapeutic agents has also become the focus of recent research. Ginsenoside Rg3 reinforces the anticancer effects when combined with cyclophosphamide [40,41,42], capecitabine [43], docetaxel [26,35], gemcitabine [44], cisplatin [32,45], doxorubicin [46], verapamil [47], and tumor necrosis factor-related apoptosis-inducing ligand (TRAIL) [48]. Additionally, ginsenoside Rg3 sensitizes cancer cells to γ-radiation by targeting the nuclear factor-kappa B (NF-κB) pathway [49], vascular endothelial growth factor (VEGF), and HIF-1α [34,50]. In vivo studies have suggested that ginsenoside Rg3 not only reduces tumor growth, but also enhances cellular immunity [51], prolongs the survival time, and improves the quality of life of mice with tumors [41].

Ginsenoside Rg3 treatment affects a broad range of signaling pathways and factors, including downregulation or deactivation of epidermal growth factor receptor (EGFR) [52,53], inactivation of NF-κB by reducing phosphorylation of extracellular signal-regulated kinases (ERK) and protein kinase B (AKT) [54], suppression of NF-κB/p65 signaling pathway [37], modulation of mitogen-activated protein kinases (MAPK) [36], down-regulation of Wnt/β-catenin signaling [27], inhibition of CXC receptor 4 (CXCR4) [55], down-regulation of AQP1 expression through p38 pathway [56], down-regulation of phosphatidylinositol 3-kinase (PI3K)/AKT family proteins and inhibitor of apoptosis protein (IAP) family proteins [22,57], reduction of histone deacetylase 3 expression and enhancement of p53 acetylation [39], inhibition of autophagy [46], suppression of anoikis resistance [58], and suppression of Warburg effect through signal transducer and activator of transcription 3 (STAT3)/HK2 pathway [59]. Furthermore, the anticancer mechanisms of ginsenoside Rg3 is related to the alteration of Bcl-2 family proteins expression, including downregulation of Bcl-2 and Bcl-xL, upregulation or activation of Bax [29,60], and activation of caspase cascade [61,62]. Another study recently found that ginsenoside Rg3 can inhibit the growth and survival of gastric cancer cells via blockade of the activity of transient receptor potential melastatin 7 channel [63], which is essential for cell survival that make it a potential target for gastric cancer treatment. Moreover, ginsenoside Rg3 also exhibits other anticancer properties, such as declination of fucosyltransferase IV (FUT4) [37,53,64], inhibition of matrix metalloproteinase 9 (MMP-9) and MMP-2 expression [65,66], blockade of hypoxia-induced epithelial to mesenchymal transition (EMT) [34,64], and attenuation of VEGF-dependent AKT/endothelial nitric oxide synthase (eNOS) signaling [67,68]. In addition, ginsenoside Rg3 induces immunogenic tumor cell death with induction of cytokine interferon-γ (IFN-γ) secretion and reduction of inflammatory cytokines IL-6, TNF-α, and TNF-β1, as well as enhances uptake of tumor cells by dendritic cells, indicating that ginsenoside Rg3 to be an effective immunotherapeutic agent [69].

#### 2.1.2. Ginsenoside Rh2

Like ginsenoside Rg3, ginsenoside Rh2 demonstrates potent anticancer effects against various cancer types, including leukemia [70,71], lung adenocarcinoma [72], colorectal cancer [73], hepatoma [74], breast cancer [74,75], ovarian cancer [76], prostate cancer [77,78], neuroblastoma [79], astroglioma [80], malignant melanoma [81], epidermoid carcinoma [82], and squamous cell carcinoma [83]. It also exhibits synergetic effects when combined with other anticancer agents, such as cyclophosphamide [84], mitoxantrone [78], and docetaxel [85]. In vivo studies have shown that ginsenoside Rh2 can efficiently inhibit tumor growth without overt toxicity when administered orally at 2–120 mg/kg body weight [77,86,87] or intravenously at 1 mg/kg body weight [88,89].

The anticancer activities of ginsenoside Rh2 and the underlying mechanisms of these activities have been intensively studied. It induces cell cycle arrest mainly in the G1 phase with concomitant downregulation of cyclin D1 and CDK4/CDK6 and increase in recruitment of p15 and p27 to cyclin D1/CDK4 and cyclin D1/CDK6 complexes; besides, ginsenoside Rh2 induces cell cycle arrest in G2 phase by downregulating cyclin B1 [75,88]. A recent study found that blockage of reactive oxygen species (ROS) by *N*-acetylcysteine or catalase inhibits Rh2-induced activation of NF-κB signaling and enhances Rh2-induced cell death, suggesting that the anticancer effect of Rh2 can be enhanced by antioxidants [73]. Bcl-2 family proteins mediate ginsenoside Rh2-induced apoptosis through downregulation of anti-apoptotic Bcl-2, Bcl-xL, and Mcl-1, and upregulation of pro-apoptotic Bak, Bax and Bim leading to activation of caspase-3 and caspase-9 [73,74]. This modulation by Bcl-2 family proteins is partially attributed to the activation of the p53 pathway [73,74,79]. Additionally, ginsenoside Rh2 induces internalization of rafts and caveolae and inactivates AKT followed by reduction of Bad and increase in Bax and Bim [82]. By increasing autophagy and by reducing β-catenin signaling, ginsenoside Rh2 eliminates cancer cells with proliferation inhibition [83,90]. Furthermore, ginsenoside Rh2 is speculated to be a potent noncompetitive *P*-glycoprotein (P-gp) inhibitor, resulting in increased cellular accumulation of compounds [91,92,93]. Nevertheless, ginsenoside Rh2 activates transforming growth factor-β1 (TGF-β1) signaling pathway though it attenuated the expression of MMP-2 and MMP-9 [88]. By recruiting histone deacetylase and by inhibiting activator protein 1 (AP-1) transcription factors, ginsenoside Rh2 can also eliminate the migratory ability of HepG2 cells [94].

#### 2.1.3. Other Ginsenosides

PPTs, including ginsenosides Rh1, Re, Rg1, and Rg2, are classified as dammarane-type ginsenosides, which possess weaker anticancer effects compared to those of the PPD counterparts [95]. Ginsenoside Rh1 exhibits concentration- and time-dependent inhibition of HepG2 cell migration and invasion by suppressing MMP1 expression through inhibition of AP-1 and MAPK signaling pathways [96]. Ginsenoside Re inhibits cell proliferation in gastric cancer cells by inducing S phase cell cycle arrest, modulating mitochondrial factors Bcl-2 and Bax, and activating caspase cascade [20]. In addition, ginsenosides Rg1 attenuates cell cycle growth arrest at G1 phase of ultraviolet B-induced HaCaT cells by modulating the protein levels involved in the p53 signaling pathway, similar to the effect of Rg2 [97]. In addition, ginsenoside Rg1 restricts TGF-β1-induced EMT in HepG2 cells [98], suppresses phorbol myristate acetate (PMA)-induced invasion and migration of MCF-7 cells by inhibiting NF-κB-dependent MMP-9 expression [99], and it inhibits the erythropoietin receptor-mediated JAK2-STAT5 signaling pathway [100].

### 2.2. Saikosaponins

Saikosaponins are a group of oleanane derivatives and the main active constituents of *Bupleuri radix* (Chaihu in Chinese), which originated in China. Saikosaponins possess a wide range of pharmacological properties, such as anti-inflammation [101], anti-virus activities [102,103], hepatoprotection [104,105], and immunomodulating activities. Saikosaponins can inhibit cancer cell proliferation and cause cell cycle arrest. Many Chinese medicine formulations containing saikosaponin A, C, and D have shown significant in vitro and in vivo anticancer effects [106,107,108]. Saikosaponin A (Figure 1c) and saikosaponin D (Figure 1d), which form a pair of epimers, are the most biologically active saikosaponins. In addition, saikosaponin B_2_ and saikosaponin C are also naturally occuring saikosaponins that demonstrate anticancer effects [109,110]. The structure-activity relationship of saikosaponins indicated that the 13,28-epoxy bridge, the orientation of the hydroxyl group, and the type of saccharide were the factors that determined the cytotoxicity of the compound in cancer cells [111].

#### 2.2.1. Saikosaponin A

It has been reported that the proliferation of cancer cells including gastric cancer [112], hepatoma [113,114], breast cancer [115], and glioma [116] can be inhibited by saikosaponin A in a concentration-dependent manner. Saikosaponin A causes G0/G1 arrest in hepatoma HuH-7 cell line [114] and breast cancer MCF-7 and MDA-MB-231 cell lines [115]. In rat C6 glioma cells, saikosaponin A demonstrated cytostatic effects and altered cell morphology at 10 μg/mL concentration and it induced cell death at 100 μg/mL concentration [116]. An experiment on HepG2 cells revealed that saikosaponin A-mediated cell growth reduction and DNA synthesis inhibition of HepG2 are possibly related to the induction of p15 and p16 mRNA expression via the PKC signaling pathway [113]. PD98059, an inhibitor of MEK, can partly reverse the increased expression of p15 and p16 proteins and growth inhibition induced by saikosaponin A, suggesting that ERK activation mediates saikosaponin A-induced HepG2 growth inhibition [113]. Following the activation of caspase-3 and alteration in expression of Bcl-2 family and C-myc, p53/p21 pathway-dependent or independent apoptosis was observed in breast MCF-7 cancer cells, and p53/p21 pathway-independent apoptosis can be observed in MDA-MB-231 cancer cells treated with saikosaponin A [115]. Saikosaponin A induces apoptosis of HCC cells by activation caspase-2 and caspase-8, cleavage of Bid and PARP, conformational activation of Bax, and decrease of IAP family members [117]. Moreover, saikosaponin A reverses MDR in MCF-7/ADR cells and HepG2/ADM cells by downregulating the expression of P-gp [118], suggesting its potential as an adjuvant therapy for clinical anticancer agents.

#### 2.2.2. Saikosaponin D

Saikosaponin D exhibits anticancer effects on various cancer cell lines, such as lung cancer [119], hepatoma [120,121,122], pancreatic cancer [120], prostate cancer [123], anaplastic thyroid cancer [124], and glioma [116]. In addition, saikosaponin D suppresses the proliferation of human hepatoma cell lines (PLC/PRF/5 and HepG2) and human pancreatic cancer cell lines (BxPC-3) by inhibiting cell growth and DNA synthesis [120]. The mechanism of the anti-proliferative effects of saikosaponin D in human non-small cell lung cancer A549 cells is similar to that in human hepatoma HepG2 and Hep3B cells [119,121]. When 0.75 mg/kg body weight of saikosaponin D was intraperitoneally injected, it reduced tumor growth, both on its own and when combined with radiation therapy [125]. Furthermore, pre-treatment with 2 mg/kg body weight of saikosaponin D prevents diethyl-nitrosamine-induced hepatocarcinogenesis and invasion in vivo [126].

Saikosaponin D can block cell cycle arrest of A549, HepG2 and ARO at G1 phase via induction of p53 expression and upregulation of p21, and downregulation of CDK2 and cyclin D1 of ARO [121,124]. Moreover, saikosaponin D inhibits proliferation and induces apoptosis in hepatocellular carcinoma SMMC‑7721 cells by suppressing the expression of cyclooxygenase (COX)‑2 and reducing the prostaglandin E2 generation by attenuating of STAT3/HIF‑1α pathway [127]. Saikosaponin D-induced apoptosis is mediated by potentiation of Fas/FasL and the increase of Bax protein in A549, HepG2, and ARO. Decrease of Bcl-xL was observed in saikosaponin D-treated HepG2 or Hep3B cells [119,121,124]. Combination with saikosaponin D can synergistically enhance the efficiency of radiotherapy in a time-dependent manner [128]. Being an endoplasmic reticulum (ER) stress inducer, saikosaponin D activates Ca^2+^/calmodulin-dependent kinase kinase/AMPK/mTOR pathway, leading to cell death [129]. In addition, saikosaponin D suppresses EMT and the expression of MMP-9 and MMP-2, inhibiting the migration and invasion abilities of cancer cells [130]. Furthermore, animal experiment on rats showed that saikosaponin D reduced the volume and weight of ARO-derived xenograft thyroid cancer model [124], and demonstrated preventive potential against DEN-induced hepatocarcinogenesis caused by suppressing of C/EBPβ and COX-2 [126].

## 3. Steroid Saponins

### 3.1. Dioscin

Dioscin (Figure 1e) is a natural steroid saponin that can be isolated from various Chinese medicines, such as *Dioscoreae rhizoma* (Shanyao in Chinese), which originates from China and *Paridis rhizoma* (Chonglou in Chinese, widespread in China and India). Dioscin exerts effects such as protection against acute chemically mediated liver injury [131], amelioration of cerebral ischemia/reperfusion injury [132], and anti-inflammatory activities [133]. Intriguingly, the anticancer potential of dioscin was effective in various cancer cells, including human leukemia K562 [134] and HL60 cells [134,135], human lung cancer A549 [136,137], NCI-H446 [137], NCI-H460 [137,138] and H1299 cells [136], human esophageal cancer KYSE510 cells [139], hepatocellular carcinoma Huh7 [140] and HepG2 cells [138], human gastric cancer SGC-7901 cells [141,142], human colon cancer HCT-116, LoVo, Caco-2, SW620, and LS cells [143], human cervix epitheloid carcinoma HeLa cells [144], human ovarian cancer SKOV3 cells [145], prostate cancer LNCaP cells [146], and human breast cancer MCF-7 cells [138], MDA-MB-231 cells, MDA-MB-453 cells, and T47D cells [147]. Moreover, dioscin exerts anticancer activities in vivo [148,149]. For example, dioscin inhibited tumor growth and angiogenesis in colon cancer C26 cell derived-tumor mouse without changing their body weight and the histology of their viscus [148]. Dioscin treatment at a dose of 300 mg/kg/day in female rats can be classified as no-observed-adverse-effect-level, and the same dose in male rats can be classified as the lowest-observed-adverse-effect level [150].

Dioscin inhibits cancer cell viabilities via various mechanisms. It causes G2/M cell cycle arrest in HCT116 cells [143] and S phase arrest ascribable to the downregulation of cyclin and CDK2 expression in C6 glioma cells [151]. Dioscin induces apoptosis via the mitochondrial pathway in HeLa [144], HL60 [152,153], SGC-7901 [141,142], HCT116 [143], KYSE510 [139], and LNCaP cells [146]. Peroxiredoxins 1 and peroxiredoxins 6 are possibly the key targets in the process of dioscin-induced apoptosis, which involves intracellular elevated ROS [139]. Dioscin increased the levels of NO and inducible NO synthase [143]. Decline of MMP and oxidative stress are mediated after dioscin uptake, leading to p38 and JNK phosphorylation and caspase cascade activation in HL60 [152], HEp-2, and TU212 cells [154]. Moreover, the amount of intracellular calcium ion increases proportionally to the concentration of administered dioscin, suggesting the involvement of Ca^2+^ in mitochondrial pathway that leads to apoptosis [141]. Sub-toxic dose of dioscin enhances TRAIL-induced apoptosis in Caki human renal cancer cells by downregulating c-FLIP_L_ [155]. Additionally, dioscin treatment considerably increases the expression of Fas, FasL, TNF-α, TNFR-1, and FADD, resulting in activation of death receptor pathways [142]. In breast cancer cells, dioscin treatment induces cell death via AIF-facilitated caspase-independent pathway and downregulation of anti-apoptotic proteins, such as Bcl-2, cIAP-1, and Mcl-1 [147]. In summary, dioscin treatment decreases mitochondrial membrane potential [139], downregulates the expression of Bcl-2 and Bcl-xL [142], upregulates expression of Bax and Bak [142], activates caspase-9, caspase-7, and caspase-3 [152], and releases cytochrome c into the cytosol [139,142,152]. It also induces DNA damage mediated by ROS [143,156]. Proteomic study shows that some differentially expressed proteins in treatment with or without dioscin are involved in oxidative phosphorylation, and in Wnt, p53, and calcium signaling pathways [143]. Nevertheless, dioscin-induced autophagy via ERK and JNK pathways possibly acts a cytoprotective mechanism against dioscin-induced apoptosis [136]. Dioscin influences on the expression of P-gp efflux pump and reverses MDR [157,158]. It restored adriamycin activity in human leukemia K562/adriamycin cells by downregulating MDR1 via a mechanism involving NF-κB signaling inhibition [159]. With the exception of 6′-*O*-methyl and the 4′′′-*O*-methyl isomers retaining part of the cytotoxicity of dioscin, other mono-*O*-methyl derivative turns out to be nearly nontoxic [160]. Furthermore, dioscin exerts anti-invasive effect, along with anti-proliferation, against breast cancer cells by enhancing GATA-binding protein 3 that regulates the transcription of several invasion-associated genes [161].

### 3.2. Polyphyllin D

Polyphyllin D (Figure 1f) is one of main active compounds isolated from *Paridis rhizoma* (Chonglou in Chinese), which has been traditionally used as an analgesic, anti-inflammatory and hemostatic drug. Its efficacy as an anti-tumor compound has long been confirmed. Polyphyllin D inhibits proliferation of cancer cells, including human leukemia K562 [162] and MDR K562/A02 cells [163], human breast cancer MCF-7 [164,165] and MDA-MB-231 cells [164], human hepatocellular carcinoma HepG2 cells [165,166], human non-small cell lung carcinoma NCI-H460 cells [165], human glioblastoma SF-268 cells [165], human glioma U87 cells [167], and human cervix epitheloid carcinoma HeLa cells [165]. Moreover, polyphyllin D eliminates MDR in R-HepG2 cells [166], inhibits P-gp-mediated daunorubicin efflux in NIH3T3 transfected cells [168], and sensitizes several ovarian cancer cell lines to cisplatin [169]. An in vivo study has shown that daily intravenous injection of polyphyllin D (2.73 mg/kg body weight) for ten days in nude mice bearing MCF-7 cells effectively reduced 50% of tumor growth in terms of tumor weight and size, causing no significant toxicity to the heart and liver of the host [164], indicating that polyphyllin D exhibited anti-cancer activity with no observable toxicity in vivo.

Polyphyllin D upregulates p21 and downregulates cyclin B1 and CDK1 in K562/A02 cells, leading to G2/M phase arrest [163]. Upregulation of typical ER stress-related proteins/genes including GRP78 and protein disulfide isomerase following polyphyllin D treatment suggested it induces cytotoxicity through a mechanism initiated by ER stress, which may further lead to apoptosis [165]. Polyphyllin D induces apoptosis through the JNK pathway in U87 cells [167]. Moreover, polyphyllin D dissipates the mitochondrial membrane potential [163,164], generates ROS [166], downregulates anti-apoptotic Bcl-2 expression [163,167], upregulates pro-apoptotic Bax expression [163,167], releases cytochrome *c* [163] and apoptosis-inducing factor [166], activates caspase-9 [164,165], caspase-4 [165], and caspase-3 [170], which cleaves PARP that associated with DNA damage and cell death [170]. The compound also inhibits migration as evaluated by wound healing assay and Transwell assays in mice lung adenocarcinoma LA795 cells [149] and Lewis lung cancer cells [171]. Polyphyllin D not only reduces cell proliferation, but also inhibits the expression of HIF-1α and VEGF mRNAs [171]. Moreover, polyphyllin D suppresses the growth of human microvascular endothelial cancer HMEC-1 cells without toxic effects and significantly inhibits cell migration and capillary tube formation [172]. Experiments using zebrafish embryos showed the defects in intersegmental vessel formation upon treatment [172], further indicating the anti-angiogenic effects of polyphyllin D.

### 3.3. Timosaponin AIII

*Anemarrhenae rhizoma* (Zhimu in Chinese) is a traditional Chinese herbal medicine that grows in China, North Korea, and Mongolia. *Anemarrhenae rhizoma* exhibits antimicrobiosis [173], antiplatelet aggregation [174,175], vascular relaxation [176], anticancer [177], anti-inflammatory [178], and memory improvement activities [179]. The aqueous extract of *Anemarrhenae rhizoma* demonstrates apoptotic effect in various cancer cell lines. Saponin components may play a major role in these effects [180]. Timosaponin AIII (Figure 1g), one of the major saponins in this herb, exhibits broad anticancer activities both in vitro and in vivo by inducing apoptosis or arresting cell cycle progress [180]. Treatment with 5 mg/kg timosaponin AIII (i.p. administration) significantly reduced tumor growth in athymic nude mice bearing HCT-15 cells with an inhibition rate of 37.3% without observable toxic effects [181]. Structure modification study on the sapogenin of timosaponin AIII showed that a piperazinyl group at C-3 would increase its cytotoxicity [182].

Timosaponin AIII can significantly inhibit cell proliferation and induce apoptosis. Particularly, it can selectively induce apoptotic cell death in breast cancer cells but not in normal cells [180]. Timosaponin AIII suppresses cell growth of human colorectal cancer cells HCT-15 via cell cycle arrest in G0/G1 and G2/M phases [181]. Besides, treatment of cancer cells with timosaponin AIII led to overproduction of ROS, reduction of mitochondrial membrane potential, suppression of mTORC1 and induction of ER stress [180], which may be associated with timosaponin AIII-mediated cell death [183]. Moreover, timosaponin AIII increases phosphorylation of JNK and p38, leading to activation of caspase-3, caspase-8, and caspase-9 activations and cleavage of PARP in a dose- and time-dependent manner [184]. Autophagy can be activated by timosaponin AIII as evidenced by induced formation of autophagic vacuoles and recruitment of LC3 [185]. Both the autophagy inhibitor 3-methyladenine and siRNA-beclin 1 enhanced timosaponin AIII-induced apoptosis [185], indicating the pro-survival potential of timosaponin AIII-induced autophagy. Moreover, timosaponin AIII reverses MDR by inhibiting PI3K/AKT signaling pathway, thereby downregulating P-gp and MRP1 expression [177]. It suppresses HGF-induced invasive activity in MDA-MB-231 cells via sustained ERK activation [186], as well as inhibits cell migration by suppressing NF-κB and COX-2 expression [187].

## 4. Discussion

Tumorigenesis is a complex process involving multifactorial interactions; thus, development of antineoplastics aiming different targets is urgently needed. Saponins are diverse and complex in structure and have shown effective anticancer potential in various cancer cell lines by inhibiting cell growth and by inducing apoptosis. Some saponins exhibit anti-metastasis [64], anti-angiogenesis [188], and anti-inflammatory [189] activities, resulting in broad application prospects of these compounds. Moreover, some saponins had been shown to reverse MDR and improve the efficacy of chemotherapy [159], suggesting the possibility of using saponins in anticancer application. This paper summarizes the anticancer activities, along with their mechanisms, of several well-known saponins isolated from Chinese medicines. The in vitro IC_50_s of these compounds are consolidated in Table 1, and the data of the treatments in vivo are presented in Table 2. The concentrations of most saponins used to demonstrate anticancer effects in vitro range from less than 1 micromolar to more than 100 micromolar. Such variation in concentration is possibly caused by the difference in cell lines, compounds, time of treatment, and evaluation methodology. Moreover, the variation in the curative effects in vivo was influenced by animal model (species, strain, gender, model, and sample size) and treatment-related factors (dosage, administration, treatment time, interval time, and combination treatment).

In addition to the aforementioned saponins, other saponins, such as gypenoside (from *Gynostemmatis pentaphylli herba*, Jiaogulan in Chinese) [200,201], ophiopogonin (from *Ophiopogonis radix*, Maidong in Chinese) [202], and astragaloside (from *Astragali radix*, Huangqi in Chinese) [203], exhibit promising anticancer properties. In particular, platycodin D (from *Platycodonis radix*, Jiegeng in Chinese), which exerts effective anti-proliferation properties that had just been well discussed, caught our sight [7]. Not only does platycodin D induce cell-cycle arrest and apoptosis, inhibit adhesion, migration and invasion abilities of cancer cells [204], and reduce tumor volume in vivo [205], but also induces autophagy by activating ERK and JNK signaling pathways [205,206]. Moreover, the combination of platycodin D with clinical medication, such as doxorubicin [207], can significantly enhance the antineoplastic efficacy of the latter. Proteomic analysis has shown that platycodin D can regulate the expression of 19 proteins in HepG2 cells [208]. These findings suggested that saponins demonstrate promising properties for antineoplastic drug development.

**Figure 2:**
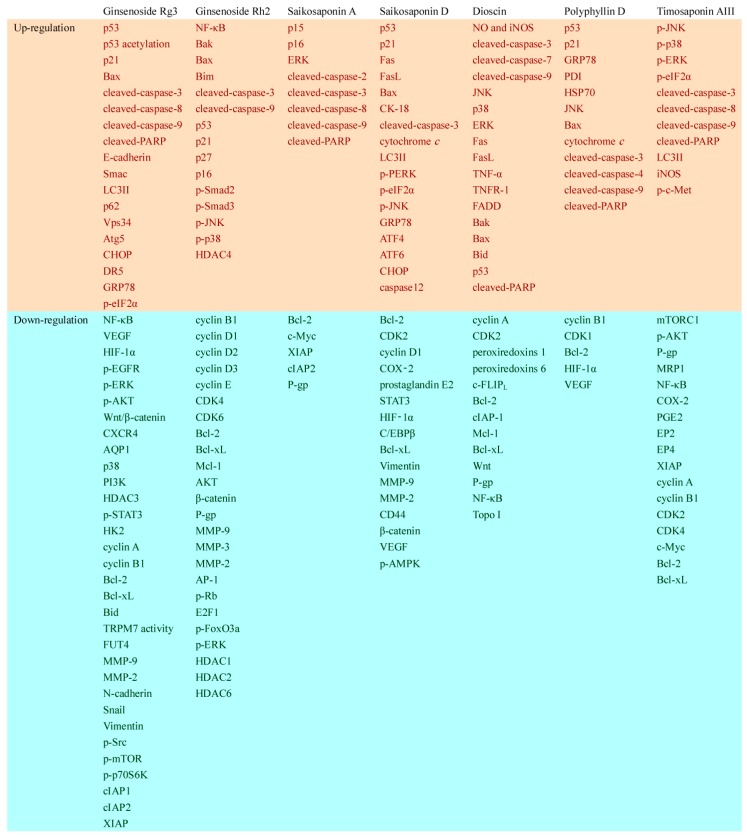
Possible mechanisms of the anticancer properties of saponins. After treatment with saponins, the upregulated protein level or activities were shown in the red columns, while the ones that be downregulated were shown in the green ones.

Saponins show concrete anticancer properties by targeting various cancer-related proteins and pathways. Figure 2 summarizes their anticancer targets and mechanisms, including cell cycle arrest, apoptosis induction, ER stress activation, migration inhibition, invasion inhibition, and MDR reversal.

Like most compounds isolated from Chinese medicines, saponins affect multiple targets, and current research has not yet succeeded in providing a clear picture of the mechanisms at work because of lack of proper technique and modeling both in vitro and in vivo. Most of the current investigations are still in vitro studies, and in vivo studies are further needed. Moreover, although some saponins found in herbal medicines and formulations have been used in clinical setting based on the theory of Chinese medicine, evidence-based clinical study remains lacking. Additionally, some issues are still needed to be addressed before saponins can be developed into anticancer agents.

Interestingly, many Chinese medicines, such as *Ginseng radix*, that are known as tonifying herbs in traditional Chinese medicine theory, demonstrate effects on immunoregulation. Saponins, as well as numerous naturally occurring polysaccharides, affect immunocytes and modulate immune function both in vitro and in vivo [209,210,211]. However, despite these findings, only few current studies have focused on the effects of saponins on cancer immunotherapy [69,212]. Immunotherapy in cancer treatment, of late years, had achieved a promising breakthrough. However, concentration of the active compound can hardly be enriched in targeted tumor tissue to the expected concentration through oral administration, leading to the speculation that saponins is effective in modulating immune response or in attenuating immune evasion rather than directly killing tumor cells in vivo. 

In terms of toxicity, saponins mainly affect the function of the gastrointestinal system, liver, kidney, heart, and genital system but only at high dosages [150,213]. At a therapeutic dosage, no significant side-effects or toxic reactions were observed in most cases in rodents (Table 2). Hence, saponins display a potential clinical use; however, despite these findings, in-depth studies and strict monitoring are still required. In addition, high dose and long-term medication of saponins should be avoided.

It is worth mentioning that, many saponins, such as PPT- and oleanolic acid-type ginsenosides, exhibit hemolytic effect, depending on their aglycones and glycosides [214]. A safer administration strategy to avoid hemolysis is oral delivery or local injection; however, the majority of saponins show low bioavailability with minimal oral absorption as a result of archenteric pH, poor membrane permeabilities, first-pass effects, and microfloral hydrolysis [215,216]. Thus, development and evaluation of a new drug delivery system for saponins is necessary. In vitro and in vivo studies on delivery systems consisting of nanoparticles, such as proliposome [217], phosphatidylcholine, and polyethylene glycol (PEG) [218] were performed, but more thorough studies are still needed.

## 5. Conclusions

In summary, saponins, a class of chemical compounds commonly found in plants and herbs and in formulations traditionally used in Chinese medicine, have been shown to exhibit promising anticancer potential. More in-depth research and development combining high-throughput and high-content screening, proteomics, biochip technology, and chemical structure modification are needed. In addition, drug delivery systems development is required to utilize this class of compound to their full potential, especially in cancer treatment. The theory of Chinese medicine and clinical practice could be also worth referring to in the process of development because of its historical use.

## Figures and Tables

**Table 1:** The anti-proliferative activities of the saponins in vitro.

Compounds	Tissue Types	Cell Lines	Effects	Reference
**Triterpenoid Saponins**				
Ginsenoside Rg3	Lung cancer	H460	IC_50_ = 392 μM/24 h	[23]
	Esophageal carcinoma	Eca-109	IC_50_ > 127 μM/48 h	[24]
	Gastric cancer	AGS	IC_50_ = 31 μM/24 h	[25]
	Colon cancer	HCT116	IC_50_ > 100 μM/24 h	[26]
	Colon cancer	HCT116	IC_50_ = 100 μM/48 h	[27]
	Colon cancer	HT29	IC_50_ = 100 μM/48 h	[28]
	Colon cancer	SW620	IC_50_ = 100 μM/24 h	[26]
	Hepatocellular carcinoma	Hep1-6	IC_50_ > 127 μM/24 h	[29]
	Hepatocellular carcinoma	HepG2	IC_50_ < 64 μM/24 h	[30]
	Renal carcinoma	786-0	IC_50_ > 127 μM/48 h	[31]
	Bladder cancer	T24R2	IC_50_ = 265 μM/48 h	[32]
	Breast cancer	MDA-MB-231	IC_50_ < 38 μM/24 h	[33]
	Ovarian cancer	SKOV3	IC_50_ = 187 μM/48 h	[34]
	Ovarian cancer	3AO	IC_50_ = 309 μM/48 h	[34]
	Prostate cancer	PC-3	IC_50_ = 85.8 μM/48 h	[35]
	Prostate cancer	PC-3	IC_50_ = 91.3 μM/24 h	[35]
	Prostate cancer	PC-3	EC_50_ = 14.1 μM/24 h	[36]
	Prostate cancer	DU145	IC_50_ = 81.5 μM/24 h	[35]
	Prostate cancer	DU145	IC_50_ = 73.1 μM/48 h	[35]
	Prostate cancer	LNCaP	EC_50_ = 8.4 μM/24 h	[36]
	Prostate cancer	LNCaP	IC_50_ = 74.7 μM/24 h	[35]
	Prostate cancer	LNCaP	IC_50_ = 62.2 μM/48 h	[35]
	Melanoma	B16	IC_50_ = 92 μM/48 h	[38]
	Melanoma	C8161	IC_50_ = 71 μM/24 h	[39]
	Melanoma	C8161	IC_50_ = 64 μM/48 h	[39]
	Melanoma	C8161	IC_50_ = 63 μM/72 h	[39]
	Melanoma	A375	IC_50_ = 64 μM/24 h	[39]
	Melanoma	A375	IC_50_ = 54 μM/48 h	[39]
	Melanoma	A375	IC_50_ = 41 μM/72 h	[39]
Ginsenoside Rh2	Leukemia	THP-1	IC_50_ = 24 μM/72 h	[70]
	Leukemia	HL-60	IC_50_ = 25.0 μM/48 h	[71]
	Leukemia	Reh	IC_50_ = 40 μM/24 h	[190]
	Leukemia	Jurkat	IC_50_ = 35 μM/24 h	[190]
	Colon cancer	HCT-116	IC_50_ = 50 μM/24 h	[191]
	Colon cancer	HCT-116	IC_50_ = 35 μM/48 h	[73]
	Hepatocellular carcinoma	HepG2	IC_50_ = 42.12 μM/24 h	[192]
	Hepatocellular carcinoma	HepG2	IC_50_ < 16 μM/24 h	[30]
	Breast cancer	MCF-7	IC_50_ = 50 μM/24 h	[74]
	Breast cancer	MCF-7	IC_50_ > 20 μM/24 h	[75]
	Breast cancer	MDA-MB-231	IC_50_ = 50 μM/24 h	[74]
	Breast cancer	MDA-MB-231	IC_50_ > 40 μM/24 h	[75]
	Ovarian cancer	KF	IC_50_ = 40 μM/5 days	[193]
	Ovarian cancer	KFr	IC_50_ = 41 μM/5 days	[193]
	Ovarian cancer	HRA	IC_50_ = 30 μM/5 days	[193]
	Ovarian cancer	KK	IC_50_ = 45 μM/5 days	[193]
	Prostate cancer	PC-3	EC_50_ = 5.5 μM/24 h	[36]
	Prostate cancer	PC-3	IC_50_ = 35 μM/72 h	[194]
	Prostate cancer	PC-3	IC_50_ = 35.7 μM/72 h	[195]
	Prostate cancer	LNCaP	EC_50_ = 4.4 μM/24 h	[36]
	Prostate cancer	LNCaP	IC_50_ = 46.7 μM/72 h	[195]
	Prostate cancer	LNCaP	IC_50_ = 17 μM/72 h	[194]
	Prostate cancer	DU145	IC_50_ = 38 μM/72 h	[194]
Saikosaponin A	Gastric cancer	AGS	IC_50_ = 34.6 μM/24 h	[112]
	Colon cancer	HCT116	IC_50_ < 20 μM/40 h	[117]
	Colon cancer	LoVo	IC_50_ < 20 μM/40 h	[117]
	Colon cancer	SW48	IC_50_ < 20 μM/40 h	[117]
	Colon cancer	SW480	IC_50_ about 20 μM/40 h	[117]
	Hepatocellular carcinoma	HepG2	IC_50_ = 13 μM/24 h	[113]
	Hepatocellular carcinoma	HepG2	IC_50_ = 23.4 μM/24 h	[112]
	Breast cancer	MDA-MB-231	ED_50_ = 6.4 μM/48 h	[115]
	Breast cancer	MCF-7	ED_50_ = 6.4 μM/48 h	[115]
	Breast cancer	MCF-7	IC_50_ = 33.3 μM/24 h	[112]
Saikosaponin D	Lung cancer	A549	IC_50_ = 10.18 μM/48 h	[119]
	Hepatocellular carcinoma	HepG2	IC_50_ = 2.63 μM/48 h	[121]
	Hepatocellular carcinoma	Hep3B	IC_50_ = 4.26 μM/48 h	[121]
	Hepatocellular carcinoma	SMMC-7721	IC_50_ > 15 μM/72 h	[127]
	Prostate cancer	DU145	IC_50_ about 10 μM/24 h	[123]
	Thyroid cancer	ARO	IC_50_ about 20 μM/24 h	[124]
	Thyroid cancer	8305C	IC_50_ about 15 μM/24 h	[124]
	Thyroid cancer	SW1736	IC_50_ about 18 μM/24 h	[124]
**Steroid Saponins**				
Dioscin	Leukemia	K562	IC_50_ = 4.7 μM/48 h	[159]
	Lung cancer	NCI-H460	IC_50_ = 18.2 μM/72 h	[138]
	Lung cancer	NCI-H446	IC_50_ about 12 μM/48 h	[137]
	Lung cancer	H1299	IC_50_ about 2.5 μM/24 h	[136]
	Lung cancer	A549	IC_50_ about 6 μM/48 h	[137]
	Lung cancer	A549	IC_50_ < 2.5 μM/24 h	[136]
	Gastric carcinoma	SGC-7901	IC_50_ = 16 μM/24 h	[141]
	Gastric carcinoma	SGC-7901	IC_50_ = 10 μM/48 h	[141]
	Gastric carcinoma	SGC-7901	IC_50_ = 4 μM/72 h	[141]
	Hepatocellular carcinoma	HepG2	IC_50_ = 8.3 μM/72 h	[138]
	Cervical cancer	HeLa	IC_50_ = 4.4 μM/48 h	[144]
	Cervical cancer	HeLa	IC_50_ = 40.2 μM/72 h	[138]
	Breast cancer	MCF-7	IC_50_ = 50.6 μM/72 h	[138]
Polyphyllin D	Leukemia	K562	IC_50_ = 0.9 μM/24 h	[163]
	Leukemia	K562/A02	IC_50_ = 0.8 μM/24 h	[163]
	Lung cancer	NCI-H460	IC_50_ = 3.0 μM/48 h	[165]
	Hepatocellular carcinoma	HepG2	IC_50_ = 7 μM/24 h	[166]
	Hepatocellular carcinoma	HepG2	IC_50_ = 3.5 μM/24 h	[165]
	Hepatocellular carcinoma	R-HepG2	IC_50_ = 5 μM/24 h	[166]
	Ovarian cancer	A2780CP	EC_50_ = 0.22 μM/72 h	[169]
	Ovarian cancer	TYKNU-CIS-R	EC_50_ = 0.25 μM/72 h	[169]
	Ovarian cancer	TYKNU	EC_50_ = 0.28 μM/72 h	[169]
	Ovarian cancer	TOV112D	EC_50_ = 0.30 μM/72 h	[169]
	Ovarian cancer	HEYA8	EC_50_ = 0.36 μM/72 h	[169]
	Ovarian cancer	TOV21G	EC_50_ = 0.37 μM/72 h	[169]
	Ovarian cancer	A2780S	EC_50_ = 0.37 μM/72 h	[169]
	Ovarian cancer	IMCC5	EC_50_ = 0.39 μM/72 h	[169]
	Ovarian cancer	OVCAR8	EC_50_ = 0.39 μM/72 h	[169]
	Ovarian cancer	M41-R	EC_50_ = 0.40 μM/72 h	[169]
	Ovarian cancer	SKOV3	EC_50_ = 0.46 μM/72 h	[169]
	Ovarian cancer	M41	EC_50_ = 0.70 μM/72 h	[169]
	Ovarian cancer	PEO1	EC_50_ = 0.81 μM/72 h	[169]
	Ovarian cancer	OVCAR2	EC_50_ = 0.85 μM/72 h	[169]
	Ovarian cancer	OVCA433	EC_50_ = 0.97 μM/72 h	[169]
	Ovarian cancer	IGROV1	EC_50_ = 0.97 μM/72 h	[169]
	Ovarian cancer	OVCAR5	EC_50_ = 1.10 μM/72 h	[169]
	Ovarian cancer	OVCA432	EC_50_ = 1.10 μM/72 h	[169]
	Ovarian cancer	OVCA420	EC_50_ = 1.27 μM/72 h	[169]
	Ovarian cancer	MCAS	EC_50_ = 1.44 μM/72 h	[169]
	Cervical cancer	HeLa	IC_50_ = 3.7 μM/48 h	[165]
	Breast cancer	MCF-7	IC_50_ = 5 μM/48 h	[164]
	Breast cancer	MCF-7	IC_50_ = 3.7 μM/48 h	[165]
	Breast cancer	MDA-MB-231	IC_50_ = 2.5 μM/48 h	[164]
	Glioblastoma	SF-268	IC_50_ = 3.0 μM/48 h	[165]
	Glioma	U87	IC_50_ = 49.4 μM/24 h	[167]
Timosaponin AIII	Colon cancer	HCT15	IC_50_ = 6.1 μM/72 h	[181]
	Colon cancer	HCT116	IC_50_ = 5.5 μM/72 h	[181]
	Colon cancer	HT29	IC_50_ = 10.3 μM/72 h	[181]
	Colon cancer	HT29	IC_50_ = 2.2 μM/72 h	[196]
	Colon cancer	SW480	IC_50_ = 13.1 μM/72 h	[181]
	Colon cancer	SW620	IC_50_ = 11.1 μM/72 h	[181]
	Hepatocellular carcinoma	BEL-7402	IC_50_ = 1.65 μM/72 h	[196]
	Cervical cancer	HeLa	IC_50_ = 9.63 μM/72 h	[196]
	Breast cancer	MDA-MB-468	IC_50_ = 1.6 μM/72 h	[196]

**Table 2:** The anticancer potential of the saponins in vivo.

Compounds	Method	Effect	Reference
**Triterpenoid Saponins**			
Ginsenoside Rg3	Daily oral administration of 20 mg/kg ginsenoside Rg3 for 18 days in C57BL/6 mice bearing Lewis lung carcinoma cells	Enhanced about 50% of survival rate and delayed about 33.3% of tumor growth without side effects	[44]
	28-day oral treatment with 100 mg/kg ginsenoside Rg3 in nude mice bearing H460 cells	Remarkably suppressed the tumor growth by decreasing the tumor volume and weight by 30%–31%	[23]
	Daily oral administration of 20 mg/kg ginsenoside Rg3 for 21 days in nude mice bearing Huh-7 cells	Reduced tumor volume for 23%.	[48]
	H22-bearing mice were injected intraperitoneally with 20(S)-Rg3 and 20(R)-Rg3 (3 mg/kg body weight) once a day for 10 days	Inhibited the 23.6% and 40.9% of tumor growth, respectively. And enhanced cellular immunity with lymphocyte proliferation and IL-2 and IFN-γ production in serum and immune organs	[51]
	Daily intra-tumor injection of ginsenoside Rg3 (3.0 mg/kg) for ten days in C57BL/6 mice bearing Hep1-6 cells	Inhibited the tumor growth by more than 50% and prolonged survival time.	[29]
	Rg3 was administered at 20 mg/kg body weight to nude mice bearing HCT116 cells daily for 3 weeks via i.p. injection	Inhibited about 70% of the tumor growth by down-regulating Wnt/beta-catenin signaling pathway	[27]
	Rg3 was injected intraperitoneally at 20 or 40 mg/kg body weight every day for 3 weeks to gallbladder cancer NOZ-bearing BALB/c nude mice	Effectively reduced tumor growth for about 60% of tumor weight	[197]
	From day 1, 5 mg/kg of Rg3 was injected via tail-vein of SKOV3-bearing mice every other day till day 30	Effectively reduced tumor growth for about 65% of tumor weight	[34]
	Daily intraperitoneal injection of 3 mg/kg ginsenoside Rg3 for 10 days in athymic mice bearing SKOV-3 cells	Prolonged 74.3% of survival time, decreased 41.9% of tumor weight, and improved life quality	[41]
	Rg3 was subcutaneously administered at 20 mg/kg body weight 3 weeks with time interval of 48 h to nude mice bearing melanoma A375 cells	Significantly inhibited the tumor volume by 52.50%	[53]
	Rg3 was administered at 20 mg/kg body weight 5 times per week for 3 weeks via i.p. injection to nude mice bearing A375 cells	Significantly reduced tumor volume by 55.65%	[39]
Ginsenoside Rh2	Daily oral administration of ginsenoside Rh2 at 20 mg/kg for 3 weeks in nude mice bearing K562 cells	Significantly inhibited the tumor volume by about 50%	[86]
	Daily gavaged with ginsenoside Rh2(S) and (R) at 2–6 mg/kg for 10 days in H22 hepatoma-bearing mice	4 mg/kg of ginsenoside Rh2(S) and (R) suppressed 42.2% and 46.8% of tumor growth without causing side effects	[87]
	Ginsenoside Rh2 was intravenously administrated at a concentration of 1 mg/kg body weight to the mice bearing reporter-carrying PC3-luc cells, twice per week for 4 weeks	The bioluminescence levels were 83.5% ± 10.5% lower than those in control group	[88]
	Daily oral gavage of 120 mg/kg ginsenoside Rh2 for 25 days in nude mice bearing PC-3 cells	Effectively delayed about 60% of tumor growth in terms of tumor volume without any overt toxicity	[77]
	Intravenous injection of 1 mg/kg ginsenoside Rh2 twice a week for 1 month in NOD/SCID mice bearing A-172 gliobalastoma cells	The bioluminescence levels were 76.8% ± 12.5% lower than those in control group	[89]
Saikosaponin D	Saikosaponin D was intraperitoneally injected at a concentration of 0.75 mg/kg body weight to the BALB/c nude mice bearing SMMC-7721 xenograft tumor, thrice a week for two weeks	Saikosaponin D treatment reduced tumor volume by 11%, while the combination with radiation therapy reduced tumor volume by 66%	[125]
	Saikosaponin D was daily intraperitoneally injected at a concentration of 2 mg/kg body weight for 17 weeks to the SD rats, starting 1 week before diethylinitrosamine induction	Saikosaponin D treatment reduced about 85% nodules at the surface of the liver without invasion to surrounding tissues	[126]
**Steroid Saponins**			
Dioscin	Orally administrated 30 mg/kg dioscin in SD rat allograft with C6 cells	The average survival time of rats in the model group was 31.5 days compared to 49.97 days in the dioscin-treated group	[151]
	Dioscin was oral administrated at the doses of 40 and 80 mg/kg body weight for 30 days to the BALB/c nude mice bearing reporter-carrying MGC-803-luc cells	Inhibited about 43% and 59% of tumor weight, respectively	[198]
Polyphyllin D	Daily administration of 2.73 mg/kg body weight through intravenous injection for ten days in nude mice bearing MCF-7 cells	Effectively reduced tumor growth for 50% in terms of tumor weight and size, given no significant toxicity in heart and liver to the host	[164]
	One week after implantation, treatment groups received their first doses of polyphyllin D (15 or 25 mg/kg body weight) and intraperitoneal administrations were carried out on 4 consecutive days per week for 4 weeks in nude mice bearing SKOV3 cells	Administration of polyphyllin D led to a 40% (15 mg/kg) and 64% (25 mg/kg) tumor growth inhibition, respectively	[199]
Timosaponin AIII	Treatment with timosaponin AIII (2 or 5 mg/kg body weight, three times/week, i.p. administration) for 4 weeks in nude mice bearing HCT-15 cells	It suppressed tumor growth without any overt toxicity. The inhibition rates of tumor size compared with control volume were 8.3% (2 mg/kg) and 37.3% (5 mg/kg)	[181]
	C57/BL mice injected with B16-F10 melanoma cells were treated with single dose of timosaponin AIII (25 mg/kg body weight) and anatomized fourteen days later	It reduced about 50% of metastasis of melanoma cells to lung in mice, and inhibited the transcription of COX-2 and NF-κB	[187]
